# Global Hippocampal Volume Reductions and Local CA1 Shape Deformations in Amyotrophic Lateral Sclerosis

**DOI:** 10.3389/fneur.2018.00565

**Published:** 2018-07-20

**Authors:** Judith Machts, Stefan Vielhaber, Katja Kollewe, Susanne Petri, Joern Kaufmann, Mircea Ariel Schoenfeld

**Affiliations:** ^1^Department of Neurology, Otto-von-Guericke University, Magdeburg, Germany; ^2^German Center for Neurodegenerative Diseases, Magdeburg, Germany; ^3^Department of Neurology, Hannover Medical School, Hannover, Germany; ^4^Leibniz Institute for Neurobiology, Magdeburg, Germany; ^5^Kliniken Schmieder Heidelberg, Heidelberg, Germany

**Keywords:** ALS, hippocampus, CA1, shape analysis, structural MRI

## Abstract

There is increasing evidence for hippocampal involvement in Amyotrophic Lateral Sclerosis (ALS). Recent neuroimaging studies have been focused on disease-related hippocampal volume alterations while changes in hippocampal shape have been investigated less frequently. Here, we aimed to characterize the patterns of hippocampal degeneration using both an automatic and manual volumetric and surface-based approach in a group of 31 patients with ALS and 29 healthy controls. Irrespective of the segmentation type, left, and right hippocampal volumes were significantly reduced in ALS compared to controls. Local shape alterations were identified in the hippocampal head region of patients with ALS that corresponds to the cornu ammonis field 1 (CA1), a region known to be involved in novelty detection, memory processing, and integration of hippocampal input and output information. The results suggest a global hippocampal volume loss in ALS that is complemented by local shape deformations in a highly interconnected region within the hippocampus.

## Introduction

Amyotrophic lateral sclerosis (ALS) is a relentlessly progressive neurodegenerative disease affecting primarily the motor neurons in the corticospinal tract and brain stem. By now it is well known that ALS pathology goes far beyond the motor system, with pathological TDP-43 inclusions being identified throughout the whole brain, including extra-motor cortical and subcortical regions (1). Clinically, patients present with a variety of different subtypes depending on the involvement of upper and lower motor neuron damage; site of symptom onset; genotype; and cognitive or behavioral impairment; for review see (2, 3). The presence of cognitive and behavioral deficits, in particular, has become a major concern, especially given the growing evidence for ALS sharing some pathological (4) and genetic (5, 6) features with the frontotemporal dementias (FTD). Although initially neglected, cognitive and behavioral impairment can occur in up to 50% of the patients (7), encompassing a range of deficits such as executive dysfunction, language impairment, apathy, memory, disinhibition, or impaired social cognition. Large population-based studies report executive and language dysfunction as the most frequently observed cognitive deficits in ALS (8–10), while the presence of memory impairment appears to be more heterogeneous (11). A recent study reported a significant verbal memory deficit in ALS, that was different from that observed in amnestic mild cognitive impairment patients (aMCI) and only explained to some extent by the coexisting executive dysfunction (12). This leaves open the question whether the observed memory deficits might be mediated by structural anatomical correlates.

While multiple brain regions have been implicated in the processing of memory, there are the structures of the medial temporal lobe, namely the hippocampus, the parahippocampal gyrus, and the entorhinal cortex, that play a crucial role in the formation of memory (13). Specifically, the degeneration of the hippocampus has been related to memory deficits in Alzheimer's Disease (AD), even in early disease stages (14). In ALS, histopathological studies reported hippocampal pathology in patients with concomitant dementia along the perforant pathway (15), which was different from AD specific hippocampal lesions (16). Imaging studies revealed structural and functional changes in the hippocampus early during disease course (17) and volume reductions (18, 19) that were related to patients' memory performance (19–21). Still, the location of these changes is yet unknown. ALS-related structural alterations within the hippocampus need to be characterized *in vivo*, as they are specifically of interest for structure-function associations.

In contrast to previous conducted volumetric studies, surface-based approaches can add to the understanding of the disease while revealing local shape deformities of a given structure. While most of the imaging literature on ALS studied the hippocampus as a single unitary entity, it needs to be accounted for that the hippocampal formation is a heterogeneous structure. It can be subdivided into different cytoarchitectonic subfields encompassing the cornu ammonis fields CA1-4, the dentate gyrus (DG), and the subiculum, or, on a functional level, it can be separated into an anterior-posterior gradient along the longitudinal axes (22, 23). To assess pathological changes of the hippocampus *in vivo*, the structure is traditionally segmented manually, a technique often considered as the “gold standard.” In the light of large data sets, multi-center studies, and longitudinal designs, and the role of the hippocampus in ALS, reproducibility of hippocampal segmentation becomes elementary, which brings in automatic segmentation approaches based on machine-learning algorithms.

In order to more specifically determine the patterns of morphologic degeneration of the hippocampus in ALS, we here investigate volumetric and shape differences using both manual and automated hippocampal segmentation *in vivo*. We hypothesized that previously reported volume reductions in the hippocampus in ALS are associated with local hippocampal subfield changes that could be detected using a surface-based approach.

## Methods

### Participants

In this retrospective study, 31 patients with ALS were recruited from the outpatient clinic of the department of Neurology at Otto-von-Guericke University. Patients were classified according to the revised El Escorial criteria (24) and disease severity was rated using the ALS functional rating scale revised (ALSFRS-R) (25). A group of 29 healthy controls without prior history of neurological or psychiatric illness served as a control group. Demographic characteristics of both groups are summarized in Table 1. The local ethics committee of Otto-von-Guericke University approved the study and all participants gave written informed consent prior to their inclusion.

**Table 1:** Demographic profile.

	**N**	**Age (years)**	**Sex (male-female)**	**Education (years)**	**ALSFRS-R**	**Site of onset (bulbar–spinal)**	**Disease duration (months)**
ALS	31	62.8 ± 13.0	21−10	13.6 ± 2.3	37.8 ± 5.4	8-23	21.6 ± 21.0
HC	29	61.8 ± 5.9	19−10	14.7 ± 3.7	na	na	na
*p*		0.35	0.86	0.44	–	–	–

### MRI acquisition

Three-dimensional, T1-weighted, structural MRI scans of the brain were acquired on a GE Signa Horizon LX 1.5T neuro-optimized magnetic resonance system (General Electric Co., Milwaukee, WI) using a standard quadrature head coil (contrast-optimized spoiled gradient-echo sequence, TE = 8 ms, TR = 24 ms; flip angle = 30°; voxel size = 1.0 × 1.0 × 1.5 mm^3^).

### Manual hippocampal volumetry and shape analysis

Prior to manual segmentation, T1-weighted images were resampled to 1 mm isotropic voxels and registered into standard space using a 6 degrees of freedom (DOF) rigid body transformation to correct for variation in head tilt using FLIRT (26), which is part of the FMRIB's Software Library (FSL) (27). Manual segmentation of the left and right hippocampi was conducted by one rater blinded to group allocation using Multitracer (http://www.loni.usc.edu/Software/MultiTracer), which is a java-based tool for anatomic delineation of grayscale volumetric images (28). The software enables the simultaneous view of the hippocampus on three orthogonal planes. The border of the hippocampus was traced from rostral to caudal in magnified images of the coronal slices while simultaneously visualizing the sagittal orientation. Delineation was performed following standardized guidelines (29) using freehand spline drawing technique that is considered to offer higher precision than the previously used voxel-by-voxel approaches (30). Segmentation included the proper hippocampus, the subiculum, and the dentate gyrus, while white matter of the alveus and fimbria were excluded. The total volume of each hippocampus was calculated by summing the areas for each plane multiplied by the slice thickness. Each hippocampus consisted of about 35 to 45 individually segmented planes.

Shape analysis was conducted using the free available shape tools software developed at the laboratory of NeuroImaging (LONI), University of California, Los Angeles (http://www.loni.usc.edu/Software/ShapeTools). The digitized points derived from manual segmentation representing the hippocampal contours in each brain slice were made spatially uniform by interpolation onto a parametric grid of 100 × 150 surface points describing the hippocampal surface of each subject (31, 32). This procedure also enables the generation of an average hippocampal surface model of all subjects, where statistical results can be mapped on. In order to assess between group differences in hippocampal shape, for each individual surface model a medial curve along the anterior-posterior axis was derived (31). Subsequently, the radial distances from the hippocampal midline to the surface boundary were computed and resulting vertices were used within a general linear model with group as a main factor and age and total intracranial volume (TIV) as covariates of no interest using the free available statistics software R (https://www.r-project.org/). The resulting *T* values for each vertex location and their corresponding *p* values were used to calculate the overall statistical significance of the radial shape differences between groups. Correction for multiple comparisons was achieved by permutation testing (*p* < 0.05) (33) using R. The maximum cluster size was determined with a flood-fill algorithm, implemented in MATLAB (http://de.mathworks.com/products/matlab/).

### Automatic hippocampal volumetry and shape analysis-FSL

Automatic segmentation of the left and right hippocampi was performed using FMRIB's Integrated Registration and Segmentation Tool (FIRST). FIRST incorporates prior anatomical information of 8 different subcortical structures separated for the left and right hemisphere by using explicit shape models (34). These models were constructed from 336 manually segmented subjects with an age range from 4 to 87 years and include both normal and pathological brains (34). Prior to segmentation, T1-weighted raw images were skull-stripped using the brain extraction tool BET (35) with the optional -B flag to reduce image bias and residual neck voxels. Skull-stripped images were linearly registered to the MNI space (1 mm MNI152 template) using 12 degrees of freedom (DOF), followed by a second stage registration to a MNI subcortical mask using FSL FLIRT (26) in order to achieve a more accurate and robust subcortical alignment (34). To obtain hippocampal volume and shape, FIRST uses a Bayesian probabilistic model that relies not only on average shape and intensity information from the training data set but also on modes of variation, that efficiently describe the ways in which the structures' shape varies most typically over a population. For each individual data set, the best shape is then determined by an iterative fitting of the model, which is described by meshes. The volumetric output from the mesh is derived by identifying the voxels through which the mesh passes (boundary voxels) and filling the area within these voxels (34). Boundary correction was done using FAST (35) tissue classification to ensure that neighboring structures do not overlap.

In addition to the volumetric information, the individual meshes can be further used for shape analysis between groups. For that purpose, the vertex locations from each subject were projected onto the surface of an average template shape as scalar values, where a positive value is outside the surface and a negative is inside. Similar to the manual shape analysis, TIV and age were included as covariates of no interest, while group was used as the between-group factor within a general linear model. Intergroup differences were assessed at each vertex location using vertex-wise threshold-free cluster enhanced (TFCE) parameters (36), which were permuted using FSL Randomise (37). Results were corrected for multiple comparisons across space (FWE < 0.05).

### Automatic hippocampal volumetry-freesurfer

In order to compare the manual segmentation with another frequently used automated segmentation tool, total hippocampal volumes for the left and right hemisphere were computed using the hippocampal subfield module (38) implemented in FreeSurfer version 6.0 (http://surfer.nmr.mgh.harvard.edu/). The segmentation algorithm is based on Bayesian inference, using a combination of manual labels obtained from *in vivo* and *ex vivo* data, including healthy controls and patients [mildly demented, Alzheimer's Disease; age range (years): 60–91 *ex vivo*, mean age: 56.3 *in vivo*] on the output from the FreeSurfer standard pipeline [“recon-all”; (39)]. Volumetric information for the whole hippocampus is than available for the left and right hemisphere, separately.

### Statistical analysis of demographic and volumetric data

Prior to statistical analysis, both manually and automatically extracted hippocampal volumes were adjusted for total intracranial volume (TIV) using the covariance method (40): adjusted hippocampal volume (HV) = original HV of each subject–α (TIV subject–mean TIV of the healthy controls), where α describes the slope of the regression between the HV and TIV in healthy controls. The adjustment of hippocampal volumes by TIV was done in order to account for head size and gender effects. For shape analysis, TIV was included as a covariate of no interest. TIV was calculated using the Gaussian mixture model within the unified segmentation approach (41) in SPM12 (http://www.fil.ion.ucl.ac.uk/spm/software/spm12/) by summing the individual tissue classes (gray matter, white matter, cerebrospinal fluid) with a threshold of 0.5 (42).

Demographic and volumetric data were plotted and visually inspected for normality of distribution as well as tested for significant deviation using Shapiro-Wilk test. Demographic data were not normally distributed and differences between groups were assessed using chi-square (gender) and Kruskal-Wallis (age, education) tests. Differences in TIV-adjusted hippocampal volume were assessed conducting fixed effects models, including group (ALS/HC) and age as fixed factors. Gender was not included in the analysis as the effect of gender on gray matter volume is fully accounted for by TIV (43). Within the patient cohort, the clinical parameter disease severity (ALSFRS-R) was normally distributed, whereas disease duration was not. In order to determine the relationship between hippocampal volumes and these clinical parameters, Spearman rank correlations were computed. The significance level for all comparisons was adjusted to *p* = 0.025 following Bonferroni correction. Differences in demographic data, hippocampal volumes, and correlation analyses were done using R version 3.4.3 (“Kite-Eating Tree”).

## Results

### Hippocampal volume

**Figure 1:**
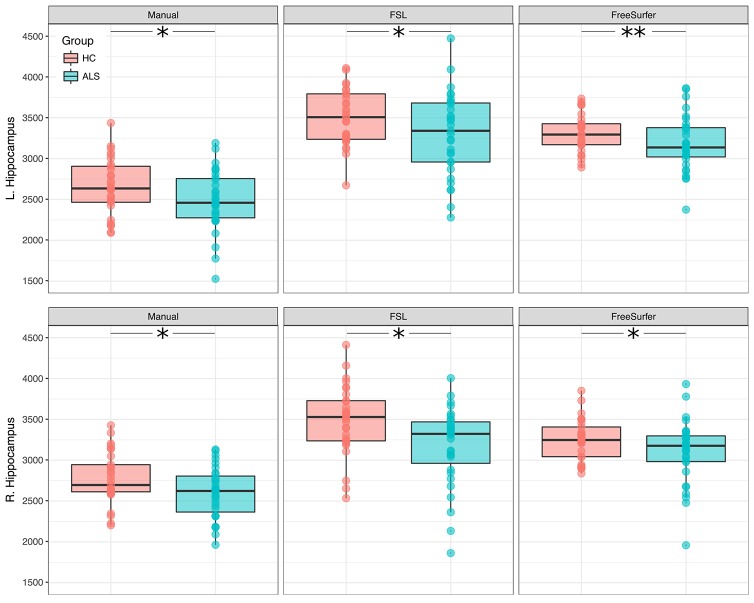
Between-group differences in hippocampal volumes (ALS and healthy controls). Boxplots display the distribution of hippocampal volumes, derived from manual **(Left)**, and automatic **(Middle, Right)** segmentation. ^*^p < 0.025; ^**^p < 0.001.

Right and left hippocampal volumes were significantly reduced in ALS compared to healthy controls when adjusting for TIV and correcting for age (RIGHT: manual segmentation: *F* = 6.17, *p* = 0.004; FSL: *F* = 5.14, *p* = 0.009; FreeSurfer: *F* = 7.09, *p* = 0.002; LEFT: manual segmentation: *F* = 4.14, *p* = 0.021; FSL: *F* = 5.74, *p* = 0.005; FreeSurfer: *F* = 11.81, *p* < 0.001). The identified volume reduction was irrespective of the segmentation type. Figure 1 displays the distribution of volumes derived from the manual and automatic segmentation. Means and standard deviations are summarized in Table 2.

**Table 2:** Means and standard deviations of hippocampal volumes.

		**Manual segmentation**			**FSL**			**FreeSurfer**		
		**ALS**	**HC**	***p***	**ALS**	**HC**	***p***	**ALS**	**HC**	***p***
Hippocampal volume [mm^3^]	Left	2,486 ± 370	2,656 ± 347	0.021	3,300 ± 507	3,501 ± 342	0.005	3,182 ± 336	3,311 ± 235	<0.001
	Right	2,600 ± 310	2,775 ± 316	0.004	3,175 ± 483	3,492 ± 426	0.009	3,108 ± 391	3,254 ± 255	0.002

**Figure 2:**
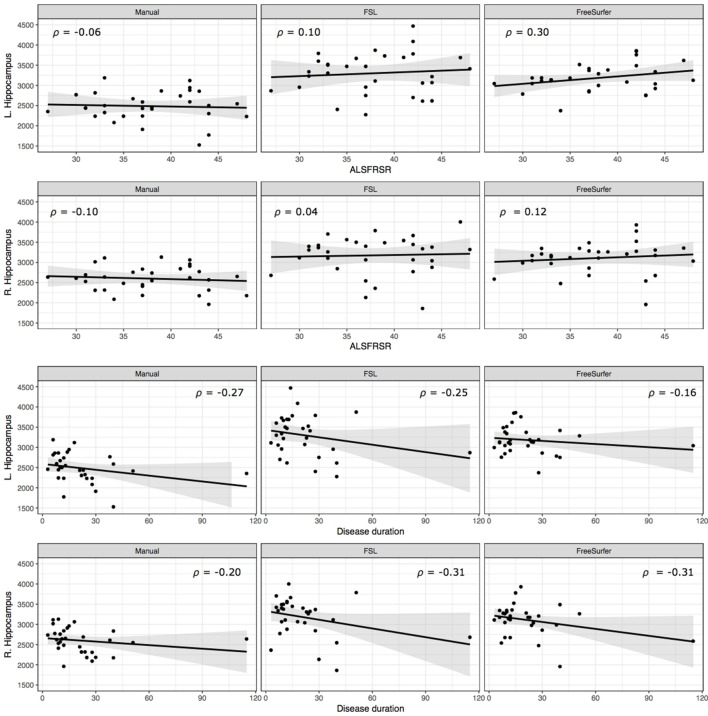
Correlation of hippocampal volume and clinical parameters. Scatterplots display the relationship between patients' ALSFRS-R score **(Top)**, disease duration **(Bottom)** and hippocampal volumes.

Bilateral hippocampal volumes were neither associated with patients' physical disability nor disease duration (Figure 2).

### Hippocampal shape

**Figure 3:**
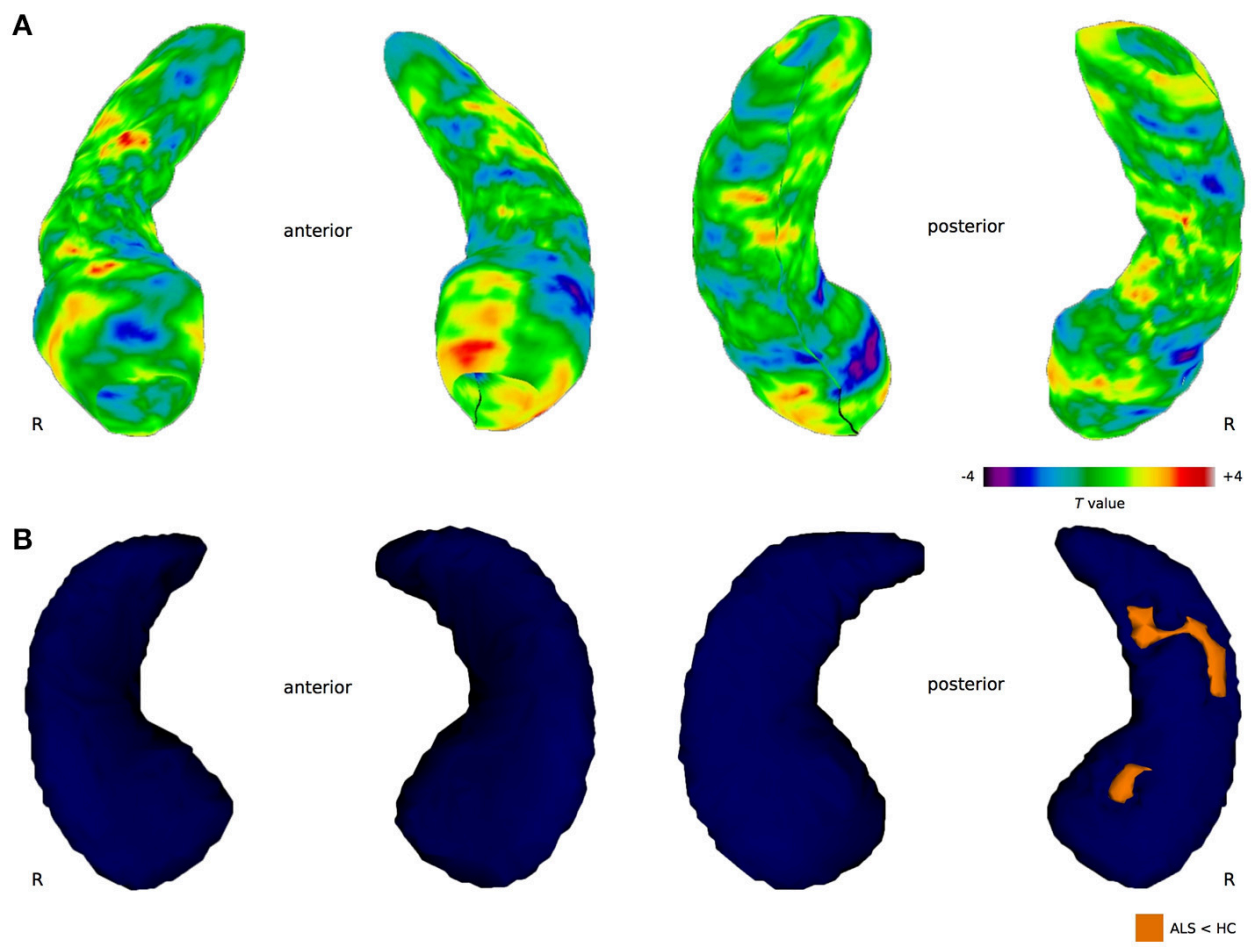
Between-group differences in hippocampal shape. **(A)** Manual shape analysis. The color bar indicates differences in radial distances between groups obtained at each hippocampal surface collection. Negative *T* values index surface shrinkage, positive *T* values index surface coves in ALS compared to healthy controls. The cluster in the left hippocampal head is significant after correction for multiple comparisons (*p* < 0.05). **(B)** Automated shape analysis. Blue color indicates the 3-dimensional template mesh, orange highlights ALS-related local shape deformations (ALS < HC). Results are corrected for multiple comparisons across space (FWE < 0.05).

Permutation tests of vertices describing the hippocampal shape derived from manual segmentation revealed no significant difference in cluster size between healthy controls and ALS patients neither for the left (cluster size = 295, *p* = 0.148) nor the right (cluster size = 106, *p* = 0.605) hippocampus. However, based on the proposed functional specialization along the longitudinal axes (22), region-of-interest (ROI) analyses were conducted for the hippocampal head, body, and tail. Permutation testing revealed local shape differences in the left hippocampal head (cluster size = 295, *p* = 0.049), but not for the right hippocampal head (cluster size = 106, *p* = 0.282) (Figure 3A). No significant clusters were found in the bilateral hippocampal body and tail.

Automated hippocampal vertex-wise analysis revealed shape deformities in the right hippocampal head and body region in ALS in comparison with healthy controls (Figure 3B) following FWE correction (*p* < 0.05). For the left hippocampal formation, no shape deformities were detected in the ALS patients compared to healthy controls at the predetermined significance threshold.

## Discussion

The current study investigated the patterns of structural degeneration of the hippocampus in ALS using a volumetric and surface-based approach. The results provide evidence for ALS-related structural alterations in the hippocampus that are characterized by global volume loss and local shape deformation in the CA1 region located in the hippocampal head.

The volumetric analysis revealed gray matter volume loss associated with ALS in the left and right hippocampus (Figure 1). The reported volume reductions were irrespective of using manual or automatic hippocampal segmentation, suggesting that automated segmentation can be safely chosen in order to investigate hippocampal volume reductions in ALS. The presented results do not point to a clinical relevant lateralization effect, but rather assume a global hippocampal volume reduction as reported by previous neuroimaging studies, where, depending on the patient cohort, either the left (18), right (19), or bilateral (44) hippocampal volumes were significantly reduced in ALS.

In addition to hippocampal volume loss, we identified ALS-related shape deformations in the hippocampal head using a vertex-wise approach based on automatic and manually segmented data. Unlike volumetric measures, the conducted shape analyses do account for the heterogeneity of the hippocampal formation, i.e., the cytoarchitectonic subfields and the anterior-posterior functional segregation along the longitudinal axis (22). Here, shape deformities were found in a region corresponding to the cornu ammonis field 1 (CA1). Both methodological approaches yielded comparable results with respect to structural alterations in the hippocampal head, although significant clusters were identified in either the left (manual) or right (automatic) hippocampus. This finding emphasizes that lateralization effects should be carefully interpreted, as they are highly dependent on the sample size, population characteristics, and methodological approach (45). In the future, such effects could be studied in a larger population of patients with ALS, which should include a group of equally distributed patients with either left or right limb onset; left or right handed; and progression of the disease on the same site as onset vs. the contralateral site.

The identified CA1-region is located in the hippocampal head, where pyramidal cells project either directly or via the subiculum to the cortex (46). This region is also known to be the primary output region of the hippocampus, while receiving its main input from CA3 neurons through Shaffer collaterals and the entorhinal cortex (47). On a functional level, CA1 is found to be critically involved in successful encoding and retrieval of long-term memory (48) and novelty detection (49). Interestingly, a recent functional MRI study investigated novelty-related hippocampal function in a group of ALS patients over the course of three months (17). Compared to healthy controls, ALS patients showed no alterations of hippocampal activation during the presentation of novel stimuli. When repeating the experiment after three months, patients with ALS showed, in contrast, a significant increase in hippocampal activity while the behavioral performance was identical to the initial measurement (17). The authors interpreted this effect as a mechanism to compensate for the beginning of structural lesions (50). The results of the present study support this hypothesis and provide a structural correlate for the reported functional alterations in the CA1 region in ALS.

In addition to the cytoarchitectonic segregation, another functional specialization within the hippocampal formation has been postulated (22, 23). Input information to the entorhinal cortex is organized in an anterior-posterior gradient (48) which is preserved throughout other hippocampal subfields (51). Within this framework, anterior parts of the hippocampus receive input from the amygdala and limbic system, while the posterior parts receive input from the visual cortex. Findings from functional MRI studies further support this organization and suggest that the anterior parts of the longitudinal axis are more engaged in emotional regulation whereas posterior regions are more involved in memory and spatial cognition (52, 53). Our findings suggest that ALS-related hippocampal pathology is primarily located in the anterior parts of the structure, thus affecting mainly the limbic system and associated functions. Recent population-based studies have shown that behavioral deficits such as apathy, stereotypies, and disinhibition are a prominent feature of ALS (54–57), and can even precede motor symptoms (58). In the light of the results presented here these behavioral deficits are likely to be associated with the identified CA1 lesion. As this was not the research question of the current study, we cannot present any behavioral data from our patient population. However, future studies will need to take care of this issue and investigate a possible relationship. Apart from behavioral deficits, Machts et al. (12) showed that memory impairment in ALS can be a feature of cognitive dysfunction, though it is different from a pure amnestic deficit frequently observed in Alzheimer's Disease (12). The findings of the current study present a structural correlate of the specific neuropsychological profile typically observed in ALS and lead to further questions regarding the role of the hippocampal formation and its connections in ALS pathology. Further studies should focus on examining regions in the brain the hippocampus is interconnected with given that ALS is now understood as a multisystem disease with neuronal degeneration likely occurring within networks, rather than in isolated regions. The hippocampus, e.g., is part of the Papez circuit, a network composed of the anterior thalamic nuclei (ATN), mammillary bodies, the cingulate cortex, and the hippocampal formation, including the parahippocampal and entorhinal gyrus that just recently has come into focus in ALS research (59). AS pointed out in the introduction, the anterior and posterior part of the hippocampus are interconnected with different cortical areas and therefore involved in completely different cognitive functions. Studying the connectivity profile of these regions would be of great interest in order to understand the contribution of the hippocampal pathology to the patients' cognitive profile.

One limitation of our study is the applied magnetic field strength of 1.5T. Although the resolution of the T1 images was high, it is difficult to exactly delineate the border between two contiguous subfields, even at higher resolution/field strength (60), and conclusions should be drawn cautiously. Nevertheless, results from histological studies confirm the differential vulnerability of CA1 neurons to excitotoxicity (61), which has been found to play a critical role in ALS pathogenesis (62), thus supporting the reported findings. While the genetic and neuropsychological status of our group is missing, the volumetric and shape deformations found in the hippocampal head match previously reported ALS-related alterations in the hippocampus (17, 20) where cognition and genetic status were included. Nevertheless, further studies investigating the hippocampus should focus even more detailed on clinical and neuropsychological subgroups of ALS, including different genetic phenotypes as well as patients with predominant memory deficits.

Taken together, the current results provide evidence for hippocampal involvement in ALS, which is characterized by global volume loss and local atrophy in the CA1 region and therefore represent a neuronal correlate for the cognitive and behavioral deficits frequently encountered in the disease.

## Author contributions

JM, SV, KK, SP, JK, MS planned and designed the study. JK and SV performed measurements. JM, JK and MS analyzed the data. JM and MS wrote the manuscript.

### Conflict of interest statement

The authors declare that the research was conducted in the absence of any commercial or financial relationships that could be construed as a potential conflict of interest.
